# Electrochemical Nickel-Catalyzed Synthesis of Unsymmetrical Diorganyl Selanes from Diaryl Diselanes and Aryl and Alkyl Iodides

**DOI:** 10.3390/molecules29194669

**Published:** 2024-10-01

**Authors:** Jona Queder, Gerhard Hilt

**Affiliations:** Institute of Chemistry, Oldenburg University, Carl-von-Ossietzky-Str. 9-11, 26129 Oldenburg, Germany; jona.queder@uni-oldenburg.de

**Keywords:** catalysis, diselanes, selanes, nickel, electrochemistry, aryl iodides, alkyl iodides

## Abstract

The synthesis of unsymmetrical diorganyl selanes was accomplished under electrochemical conditions in an undivided cell utilizing a magnesium cathode and a carbon anode made out of aryl and alkyl iodides and diselanes. This electrochemical cross-electrophile coupling (eXEC) was accomplished using a simple nickel catalyst formed in situ out of Ni(acac)_2_ and 2,2′-bipyridine in DMF at ambient temperatures. The reaction showed good functional group compatibility, and heteroaryl iodides, such as thiophene or pyridine derivatives, were well accepted.

## 1. Introduction

**Figure 1:**
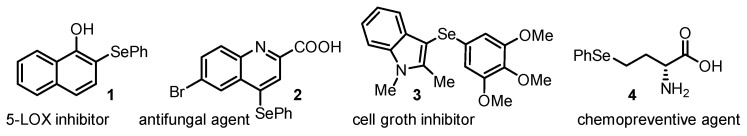
Biologically active unsymmetrical diaryl selanes and a biologically active alkyl–aryl-selane.

A number of unsymmetrical diorganyl selane compounds exhibit useful (= non-lethal) biological applicability [1,2], such as phenylselenyl-substituted naphthol derivative **1**, which is applied as a 5-LOX inhibitor (Figure 1). Phenylselanyl-substituted quinoline derivative **2** is an antifungal agent, whereas multiple-substituted 3-indolyl selane **3** is used as an efficient cell growth inhibitor. Besides the well-known α-amino acids selenocysteine and selenomethionine, aryl-alkyl-selanes, such as α-amino acid **4,** exhibit useful biological activity; **4** acts as a chemopreventive agent.

Also, chiral unsymmetrical diaryl selanes and alkyl–aryl-selanes are of synthetic value—for example, in the selenium-catalyzed oxidative cyclization of β,γ-unsaturated carboxylic acid, as reported by Maruoka in 2016 [3]. Recently, an excellent review concerning the application of selenium compounds to asymmetric synthesis was published by Back [4].

**Scheme 1:**
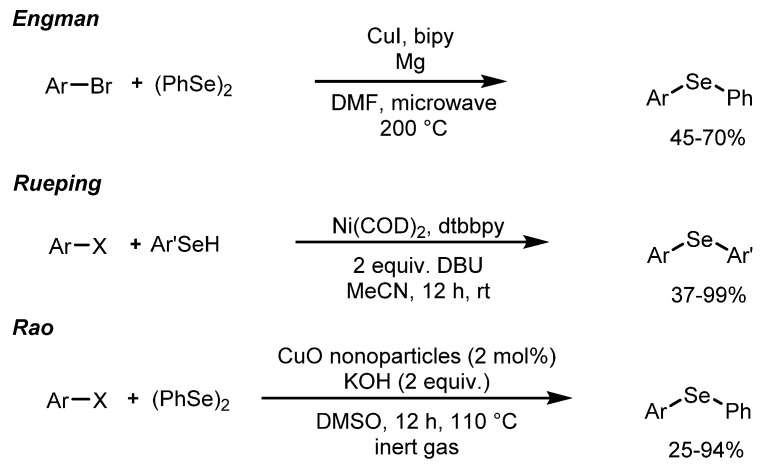
Recent access routes for unsymmetrical diorganyl selanes.

For the synthesis of such unsymmetrical selanes, the recent focus has been on the investigation of cross-coupling methodologies [5,6]. Transition metal-catalyzed coupling reactions, in most cases, rely on high temperatures, as shown by Engman [7]; the application of strong bases, as exemplified by Rueping [8]; photochemical activation [9,10]; or more sophisticated approaches, like the use of catalytically active nanoparticles, as described by Rao [11] (Scheme 1).

**Scheme 2:**
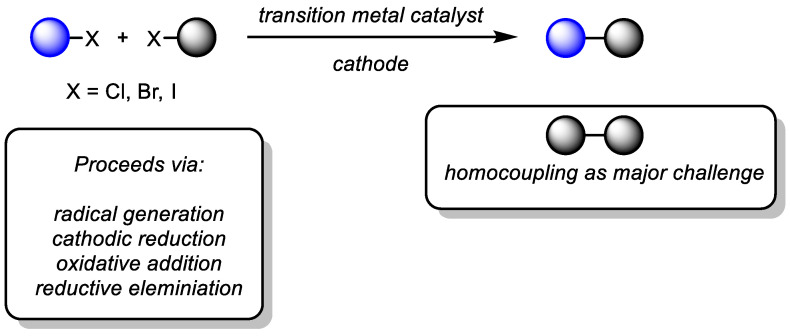
General concept of electrochemical cross-electrophile coupling (eXEC).

To overcome the challenges faced in classical cross-coupling chemistry, electrochemically driven cross-electrophile coupling (eXEC) has emerged as a new methodology [12,13,14]. The electrochemical coupling of two electrophiles, formally accompanied by a two-electron reduction process, facilitates the desired cross-coupling via radical pathways [15], making the disadvantages of the classical pathways mentioned above obsolete (Scheme 2) [16]. A crucial point in this strategy is that the redox potentials of the two electrophiles are different so that selective reduction can occur. Naturally, electrochemical activation of one of the starting materials can either be accomplished by potentiostatic electrolysis with a fixed redox potential with respect to a reference electrode or by galvanostatic electrolysis at low current densities. Therefore, the larger the difference in the reduction potential, the more selective the activation process.

The idea of an eXEC reaction pathway for mild synthesis of unsymmetrical diorganyl selanes inspired us to initiate an investigation of an eXEC process where electrochemically generated selanyl radical species could react with aryl or alkyl iodides under nickel catalysis. The focus of the proposed reaction was set to realize the synthesis of the diorganyl selane under mild reaction conditions with broad functional group tolerance from easily accessible starting materials.

## 2. Results and Discussion

As easily accessible test substrates, we chose 4-iodotoluene (**5a**) and diphenyl diselane (**6**) for the formation of the unsymmetrical phenyl-4-tolylselane (**7a**, Table 1). 

Optimization of the nickel-catalyzed formation of **7a** was performed with respect to the type and amount of the supporting electrolyte and the nickel precursor, the additive ligand, the solvent, and the typical electrochemical parameters, such as the cell type, the anode/cathode material, and the current required to reach the highest conversion and yield of product **7a**. Selected examples of the optimization process are given in Table 1 (for additional test reactions, see the Supporting Information). The individual optimized parameters were then combined, and the optimized process consisted of Ni(acac)_2_/bipy-mediated electrochemical transformation with a magnesium sacrificial anode and an RVC (Reticulated Vitreous Carbon) cathode in an undivided cell, utilizing DMF as the solvent and LiClO_4_ as the supporting electrolyte at room temperature and under a low current (3 mA), utilizing 2 *F* of electricity in an inert atmosphere to afford the desired product at a 91% yield. 

During the optimization process, we checked the above-mentioned individual parameters, while some parameters were not yet optimized (see the Supporting Information). However, typical trends could be observed, and the most important set of information is given in Table 1. 

As supporting electrolytes, tetrabutylammonium salts facilitated the reaction but were inferior to lithium perchlorate, which gave the highest yield of **7a** when a 0.5 M solution of LiClO_4_ was used (entries 1–3). Several nickel salts could be applied as the catalyst precursor (entries 4–6,) among which Ni(acac)_2_ proved to give the highest conversion when 25 mol% (Table 1) was used. 

But for no obvious reason, the yield dropped significantly when the catalyst loading was only slightly reduced or increased (entries 7/8). Interestingly, several bipyridine derivatives (see the SI) and phenanthroline (entry 9) resulted in the formation of active nickel catalysts, whereas phosphine ligands, such as dppe (entry 10), inhibited the desired reaction completely. 

**Scheme 3:**
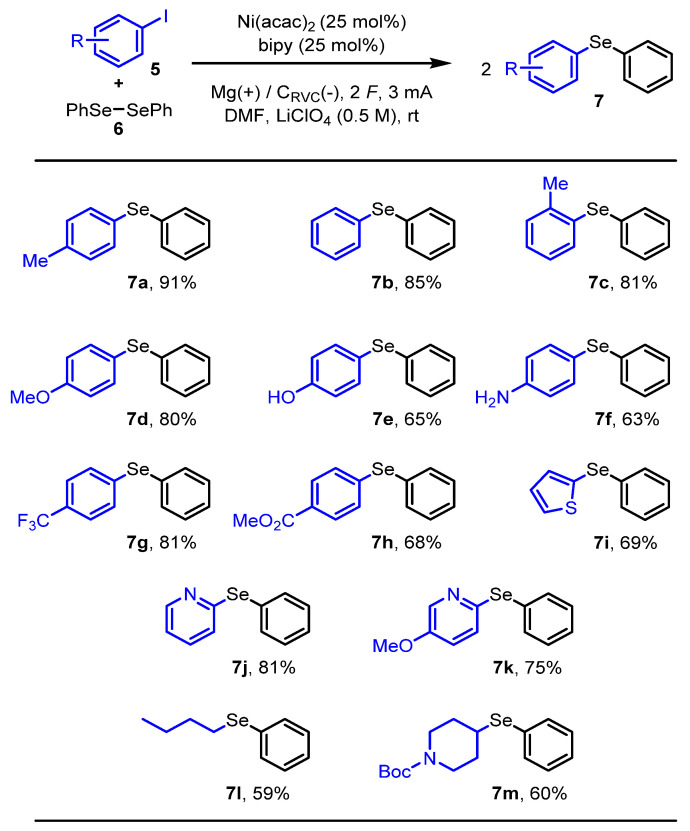
Scope of the nickel-catalyzed electrochemical synthesis of unsymmetrical diaryl selanes.

Solvents with an amide subunit, such as DMF (Scheme 3) and DMA (entry 11), gave good results, while acetonitrile (entry 12) was less suited as the solvent. Slightly acidic solvents, such as trifluoroethanol, inhibited the reaction completely (entry 13). The reaction is best performed in an undivided cell (with a 5 mm electrode distance), while in a divided cell (P4 frit), only traces of the product are found, and no product is formed in a quasi-divided cell (entries 14/15). These results are of interest because they show that both electrode reactions are needed for the overall process to be successful. As cathode material, graphite, glassy carbon, or a Mg plate can be used (entries 16–18). The anode reaction is best performed using a Mg anode or with much less efficiency using a Zn anode (20%), but the reaction fails when a Pt anode is used (entries 19−20). The electrolysis should be stopped after the consumption of 2.0 *F*, resulting in the best results, while less or more electricity results in lower yields (entries 21/22).

With the optimized reaction conditions hitherto at hand, the application of several selected functionalized iodobenzene derivatives (**5**) with diphenyl diselane (**6**) was investigated for the synthesis of unsymmetrical selanes **7**. The results of this investigation are summarized in Scheme 3.

Under the optimized conditions, the use of iodobenzene and alkyl-substituted iodobenzenes led to the formation of **7a**–**7c** at good isolated yields ranging from 80 to 91%. Of considerably more interest were heteroatom-functionalized iodobenzene derivatives of type **5** in terms of determining the limiting factors based on their electronic nature. Therefore, we investigated alkoxy-functionalized iodobenzene derivatives, such as 4-iodanisole and 4-iodophenole, as typical electron-rich arenes. The desired products **7d** and **7e** were formed at 80% and 65% yields, respectively. With a similar efficiency, 4-iodoaniline was converted into product **7f**, which was isolated at a 63% yield. Substrates with electron-withdrawing substituents on their iodoarene moiety were also reactive under the reaction conditions, and the trifluoromethyl (**7g**) and methyl benzoate derivatives (**7h**) were produced at moderate to good yields of 81% and 68%, respectively. Heterocyclic iodoarenes were then investigated, and the 2-thienyl, 2-pyridinyl, and 4-methoxy-2-pyridinyl moieties were well accepted in this process. The desired products **7i**–**7k** could be isolated again at moderate to good yields, ranging from 69 to 81%. Finally, alkyl iodides were also investigated, and as the test substrate, a simple primary alkyl iodide, such as butyl iodide, was reacted with diphenyl diselane. The desired product **7l** was isolated at a moderate yield (59%), but this result indicated that the reaction was not strictly limited to aryl iodides. Last but not least, the secondary Boc-protected piperidinyl iodide was converted into **7m**, and the product was isolated at a 60% yield. In this reaction, a small portion (5–10%) of a Boc-deprotected piperidine selane was also observed but could not be isolated in its pure form. 

According to these results, electron-deficient, electron-neutral, and electron-rich aryl iodides could be transformed into the desired selanes at moderate to good yields. Also, heteroaryl iodides were well accepted as starting materials, as were alkyl iodides, showing few restrictions concerning the application to forming selanes of primary and secondary alkyl derivatives.

**Scheme 4:**
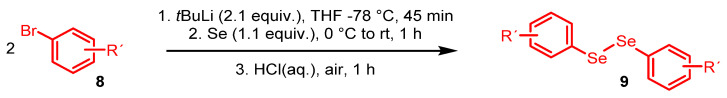
Synthesis of bis-functionalized diaryl diselanes.

The modification of diaryl diselanes is more complex since very few diaryl diselanes are commercially available. Diselanes **9** must be prepared from suitable aryl bromides **8** in multi-step synthesis, as is outlined in Scheme 4. The synthesis of diselanes was described by us in a preliminary study [17] and therefore should only be discussed briefly. Aryl bromide **8** is treated with excess *t*BuLi at a low temperature before elemental grey selenium is added and the mixture is stirred for 1 h. The solution is then acidified, and the selenole intermediate is oxidized by air at room temperature to yield the corresponding diselane **9**, which is then purified by column chromatography.

**Scheme 5:**
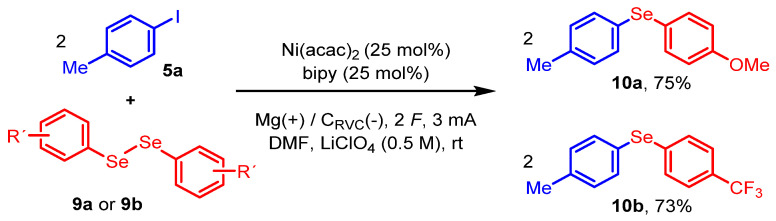
Nickel-catalyzed synthesis of unsymmetrical bis-substituted and functionalized diaryl selanes.

Thereby, we obtained two diselane candidates with different electronic natures, with diselane **9a** bearing a methoxy group in the 4-position and diselane **9b** having an electron-abstracting 4-trifluoromethyl group. These diselanes were then subjected to the optimized reaction conditions and reacted with 4-iodotoluene **5a** as an electron-neutral test substrate. The results of these two reactions are given in Scheme 5.

Both products (**10a** and **10b**) were obtained at a good yield (>73%) and showed that the nickel-catalyzed synthesis of unsymmetrical diaryl selanes is capable of accepting all sorts of functionalized iodo(hetero)arenes and electron-rich and electron-deficient diselanes as starting materials. 

**Scheme 6:**
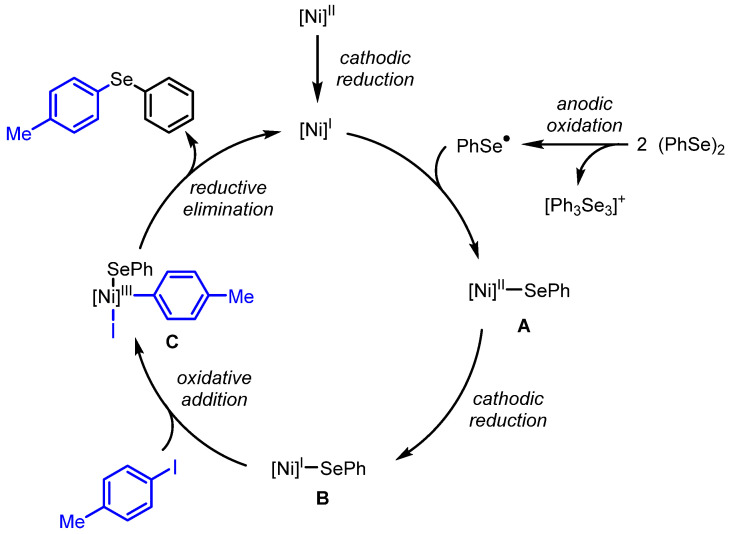
Proposed mechanism of the nickel-catalyzed synthesis of bis-functionalized diaryl selanes. The ligands are omitted for clarity.

The observations made during our examination of the reaction led us to a mechanistic interpretation consistent with one of the most prominent mechanisms for XEC reactions [18,19]. It is proposed that the modified radical chain mechanism, which depicts the net coupling of two electrophiles, proceeds via anodic radical formation and electrochemical manipulation of the oxidation state of the Ni (II) intermediates. As demonstrated in recent research [20], Ni (I) (bipy)halogen complexes are of noteworthy stability, whereas the corresponding Ni (0) species and Ni (II) species tend to comproportionate with Ni (I), leading us to believe that the latter is the resting state of the catalyst [21]. Once the Ni (II) precursor has been reduced, complex **A** is formed by trapping the anodically generated selanyl radical (Scheme 6). Following cathodic reduction, intermediary Ni (III) complex **C** is formed through following the oxidative addition of iodobenzene to Ni (I)SePh complex **B**. 

In a classical radical chain mechanism, Ni (I) intermediate **B** would react with one of the electrophiles to form a reactive radical [22] and a Ni (II) species. We surmised that conducting the reaction in a divided cell, where the generation of the selanyl radical is anodically precluded, should therefore still result in the formation of the desired product. Subsequent experimental investigations indicate a markedly reduced yield (12%), suggesting a relatively slow rate of radical formation and the absence of a requirement for Mg ions in the reaction process. When the rate of radical formation was controlled electrochemically [23] in an undivided cell, the yield was increased to 91%, presumably by bypassing the previously described mode of radical formation through anodic oxidation of diphenyl diselane. To rule out the premature oxidation of diselane by non-electrochemical oxidation on the surface of the Mg electrode, a control experiment was carried out using Mg turnings. Even after two days of reaction and additional heating, no formation of the desired product was observed.

## 3. Materials and Methods

### 3.1. General Information

To work under an inert gas atmosphere, the solvents used were purchased water- and oxygen-free from Thermo Fischer Scientific and used in an Acroseal system. The solvents used for column chromatography were purified by distillation prior to use. All chemicals and reagents were purchased from commercial suppliers without further purification, if not stated otherwise, or were prepared according to the known procedures from the literature. Where water- or air-sensitive compounds were used, the experiments were carried out in oven-dried glassware using conventional Schlenk techniques under an argon atmosphere. NMR spectroscopy: The multiplicities of individual detected signals were given as they could be observed, not as theoretically expected. s (singlet), d (doublet), t (triplet), and m (multiplet) signals are indicated, as are their combinations. ^1^H NMR spectra were measured with an Avance 500 (500 MHz) from Bruker at 305.0 K. The samples were measured in deuterated chloroform (CDCl_3_). A pre-set pulse program was used for all the measurements. ^1^H NMR: (CHCl_3_: *δ* = 7.26 ppm); ^13^C NMR: (CDCl_3_: *δ* = 77.00 ppm); ^19^F NMR: (CCl_3_F: *δ* = 0.00 ppm); ^77^Se NMR: (MeSe-SeMe: *δ* = 275.00 ppm). Measurements of the ^77^Se and ^19^F spectra were taken using external standards. IR spectra: All the IR spectra were obtained on an IRSpirit from Shimadzu with the QATR-S Single Reflection ATR Accessory. The position of the absorption bands is indicated in wavenumbers (cm^−1^). Column chromatography: Column chromatography, used for purification of the products, was carried out with silica gel 60 (grain size: 40–63 μm) from Macherey Nagel. Thin-layer chromatography was carried out on Merck TLC plates coated with silica gel 60 F_254_ with a fluorescence indicator. For the detection of the ultraviolet light signals (λ = 254 nm), GC analysis or heating the plate after it had been dipped in a KMnO_4_ solution was used. Melting point determination: The melting point was determined in glass capillaries on a Mel-Temp from Laboratory Devices, Cambridge.

### 3.2. The General Procedure

An oven-dried undivided cell was charged with diphenyldiselane (0.5 equiv.), the organo halogen compound (1.0 equiv.), bipyridine (0.25 equiv.), nickel bis-acetylacetonate (0.25 equiv.), and lithium perchlorate (3.75 equiv.). The solids were purged with inert argon gas thrice and left in vacuo for 30 min. Afterwards, anhydrous DMF (7 mL) was added, and the programmed electrolysis was performed (3 mA, 2.0 *F*, Mg (+)/C_RVC_ (-), 1500 rpm) under an argon atmosphere. After complete consumption of the educt was confirmed via GC/MS, the resulting blackish mixture was filtered over silica gel (eluent Et_2_O) and washed with water (50 mL) once. The aqueous phase was extracted with Et_2_O (50 mL) once, and the combined organic phase was dried over MgSO_4_. The solvents were removed under reduced pressure, and the crude product was purified by flash column chromatography.

### 3.3. Characterization Data of Novel Compounds

#### 5-Methoxy-2-(phenylselanyl)pyridine (**7k**)

Yield: 75%, colorless oil. R*_f_* = 0.33 (*n*-pentane:Et_2_O = 3:1) ^1^H NMR (500 MHz, CDCl_3_): *δ* = 8.36 (d, *J* = 2.32 Hz, 1H), 7.74 (dd, *J* = 8.54, 2.35 Hz, 1H), 7.36−7.34 (m, 2H), 7.25–7.21 (m, 2H), 6.71 (d, *J* = 8.60 Hz, 2H), 3.95 (s, 3H) ppm. ^13^C{^1^H} NMR (125 MHz, CDCl_3_): *δ* = 164.1, 152.4, 145.3, 132.2, 131.3, 129.4, 127.0, 118.0, 112.3, 53.8 ppm. ^77^Se{^1^H} NMR (95.4 MHz, CDCl_3_): *δ* = 364.7 ppm. IR (ATR): λ^−1^ = 3057, 2981, 2941, 2868, 2840, 1577, 1557, 1474, 1427, 1358, 1300, 1278, 1244, 1178, 1157, 1122, 1092, 1067, 1018, 1004, 998, 925, 825, 731, 688, 667, 647, 628, 531, 497 cm^−1^.

All the other compounds are known in the literature, and the analytical data are in accordance with the published data. For details, see the Supporting Information.

In conclusion, the electrochemical eXEC reaction was designed to utilize readily available aryl iodides and diselanes as electrophiles, where electron transfer generates selanyl radicals, and in the presence of a nickel catalyst, the radicals are trapped, and further reduction of the nickel–selanyl complex facilitates the oxidative addition of the aryl iodide. Thereby, several unsymmetrical diaryl selanes and alkyl–aryl-selanes were generated at moderate to good yields. Moreover, the process accepted electron-rich as well as electron-deficient diaryl-diselanes as the starting materials, which resulted in the formation of more complex selanes at good yields.

## Figures and Tables

**Table 1:**
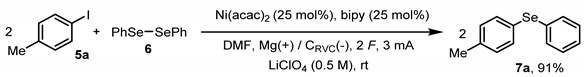
Optimization results for the nickel-catalyzed formation of unsymmetrical diaryl selane **7a**.

Entry	Change to the Conditions	Yield
1	Bu_4_NPF_6_ as supporting electrolyte	58%
2	LiClO_4_ (0.5 M) as supporting electrolyte, glassy carbon cathode	66%
3	LiClO_4_ (0.24 M) as supporting electrolyte, glassy carbon cathode	48%
4	NiCl_2_·6H_2_O as nickel catalyst precursor	62%
5	NiBr_2_ as nickel catalyst precursor	52%
6	NiI_2_ as nickel catalyst precursor	57%
7	Ni(acac)_2_ (20 mol%)	43%
8	Ni(acac)_2_ (30 mol%)	44%
9	1,10-phananthroline as ligand	46%
10	1,2-(diphenylphosphino)ethane (dppe) as ligand	2%
11	Dimethylaminoacetamide (DMA) as solvent	50%
12	Acetonitrile (MeCN) as solvent	36%
13	2,2,2-trifluoroethanol as solvent	0%
14	Divided cell (P4 frit) (2 *F*)	12%
15	Pseudo-divided cell (Pt wire anode)	0%
16	Glassy carbon cathode	57%
17	Magnesium cathode	56%
18	Platinum cathode	0%
19	Zinc anode	20%
20	Platinum anode	0%
21	1.5 *F* of electricity	42%
22	2.5 *F* of electricity	27%
23	None	91%

Conditions: Ni(acac)_2_ (25.7 mg, 0.1 mmol), bipy (15.6 mg, 0.1 mmol), 4-iodotoluene (87.2 mg, 0.4 mmol), LiClO_4_ (106 mg), and (PhSe)_2_ (62.4 mg, 0.2 mmol) were weighed in an undivided cell, anhydrous DMF (3 mL) was added, and the solution was electrolyzed (Mg (+)/C_RVC_ (-), 2 *F*, 3 mA) under an argon atmosphere. The yield was determined via GC (FID) using mesitylene as the internal standard.

## Data Availability

All the relevant data regarding this investigation can be found in the Supporting Information.

## Supplementary Materials

The following supporting information can be downloaded at https://www.mdpi.com/article/10.3390/molecules29194669/s1. The characterization data on the known compounds and the ^1^H and ^13^C NMR spectra are available online.
