# Supplementary material for: Electrochemical Nickel-Catalyzed Synthesis of Unsymmetrical Diorganyl Selanes from Diaryl Diselanes and Aryl and Alkyl Iodides
Source: Molecules. 2024 Oct 1;29(19):4669. doi: 10.3390/molecules29194669 (PMC11477634; doi:10.3390/molecules29194669)
Supplement: Supplementary file 1 [file molecules-3203351-supplementary.pdf › molecules-3203351-supplementary.pdf]

Reviewer 1:

Comments and Suggestions for Authors

The authors described an electrosynthesis method for the syntheis of unsymmetrical diaryl selanes from diselenides and aryl iodides.

1. The Rueping method in Scheme 1 middle does not use aryl diselenides but aryl selenols as the selenium source. This is important to the reaction as the latter serves as a nucleophilic after deprotonation. It's also more common to cite the name of the corresponding author in the scheme.

Changed – page 1/2 line 34 and Scheme 1

2. Table 1 and Scheme 3 can be merged into one table. Correct compound numbering should be given to the diaryl selane in Table 1 title.

Done – see page 3 Scheme 3 was merged with Table 1 and the numbers of the schemes revised.

3. What's the difference between entry 2 and 23 in Table 1?

Changed – see Table 1

4. The proposed reaction mechanism is a net-redox neutral pair electrolysis describe the reaction being described as eXEC. An anodic oxidation of PhSeSePh is necessary in each turnover. Can PhSeSePh get oxidized before Mg on a Mg anode? Does cyclic votammetry of diselenides support this hypothesis?

We added additional comments to support our proposed mechanism. Page 6 and 7

5. The proposed reaction mechanism is not supported by proper citation. Could the reaction proceed through a similar mechanism to the Cu/Mg system by Taniguchi (Synlett 2003, 6, 0829) but with Ni/cathodic reduction/Mg^{2+}?

See above…

6. Typo: Line 219, "educt".

I can not identify a typo!

While the synthetic method is solid and the experimental studies were conducted rigorously, the proposed mechanism disagrees with the reaction being classified as an eXEC reaction. Thus, I wouldn't recommend the publication of the manucript in the

current status but would encourage the authors to revised the proposed mechanism and design control experiments for mechanistic studies.

We added additional comments to support our proposed mechanism. Page 6 and 7

Reviewer 2:

1. The authors have suggested a mechanism without any citation to the literature and providing acceptable scientific documents about the formation of suggested intermediates. Thus, this part must be improved. Suitable references containing similar mechanism must be cited. For the formation of radicals and intermediates the authors must provide some evidences and study their presence and the rate of their formation.

Changed – see above.

2. Compare related work on the electrochemical efficiency of Diorganyl-Selanes

See introduction

3. Some topic of metal-catalyzed Synthesis could be addressed, such as

Chem. Commun., 2022, 58, 6653–6656; Polym. Chem., 2022, 13, 2351–2361; J. Org. Chem. 2019, 84, 14627−14635; Org. Lett. 2020, 22, 8086−8090.

These references have nothing to do with the manuscript – I suppose a copy/paste error by the reviewer?

4. I suggest the author may add the DFT to support the current result on the mechanism.

NO!!!! Definitively NOT!

Editor:

Review 2 recommended that you include references to some works in your manuscript. Before including any references recommended by reviewers please critically analyze them to ensure that their inclusion would enhance your manuscript. If you believe these references are not necessary you should not include them.

The conclusion section was moved to the end of the manuscript
